# Transcriptomic profiling of bovine IVF embryos revealed candidate genes and pathways involved in early embryonic development

**DOI:** 10.1186/1471-2164-11-23

**Published:** 2010-01-11

**Authors:** Wen Huang, Brian S Yandell, Hasan Khatib

**Affiliations:** 1 Department of Dairy Science, University of Wisconsin-Madison, Madison, WI 53706, USA; 2 Departments of Statistics, Horticulture and Biostatistics & Medical Informatics, University of Wisconsin-Madison, Madison, WI 53706, USA

## Abstract

**Background:**

Early embryonic loss is a large contributor to infertility in cattle. Although genetic factors are known to affect early embryonic development, the discovery of such factors has been a serious challenge. The objective of this study was to identify genes differentially expressed between blastocysts and degenerative embryos at early stages of development.

**Results:**

Using microarrays, genome-wide RNA expression was profiled and compared for in vitro fertilization (IVF) - derived blastocysts and embryos undergoing degenerative development up to the same time point. Surprisingly similar transcriptomic profiles were found in degenerative embryos and blastocysts. Nonetheless, we identified 67 transcripts that significantly differed between these two groups of embryos at a 15% false discovery rate, including 33 transcripts showing at least a two-fold difference. Several signaling and metabolic pathways were found to be associated with the developmental status of embryos, among which were previously known important steroid biosynthesis and cell communication pathways in early embryonic development.

**Conclusions:**

This study presents the first direct and comprehensive comparison of transcriptomes between IVF blastocysts and degenerative embryos, providing important information for potential genes and pathways associated with early embryonic development.

## Background

The decline in reproductive efficiency in high producing dairy cows has become a worldwide challenge to the dairy industry and scientific community [1]. Successful fertilization and normal embryonic development are two main components of fertility. There is a growing concern about early embryonic loss, which accounts for a large proportion of infertility, particularly in high-producing cows [2]. Moreover, the bovine has become an increasingly popular animal model for studying development of human embryos because of similar biochemical processes in these species [3,4]. However, little has been understood concerning the mechanisms underlying proper early embryonic development in cattle.

Genome-wide expression profiling by microarrays has proved a highly effective tool for high throughput analysis of transcriptomes of tissues, cell lines, or any biological mRNA pools, usually across different stages, conditions, or treatments. Indeed, a number of studies have utilized microarrays to understand the dynamics of gene expression during early embryonic development. For example, Misirlioglu et al. [5] and Kues et al. [6] investigated the dynamics of gene expression and defined subsets of genes regulated during preimplantation development of bovine embryos, particularly those related to embryonic genome activation. In addition, using a cDNA microarray consisting of 932 bovine ESTs, between in vitro- and in vivo-cultured blastocysts of varying quality, Corcoran et al. [7] were able to identify 384 differentially-expressed genes that were believed to affect subsequent survival and pregnancy. However, no study has directly investigated changes in gene expression associated with abnormal early embryonic development or growth retardation of embryos.

An in vitro fertilization (IVF) system has been previously established in our laboratory to identify genetic markers for fertility traits in cattle [8-10]. Specifically, the developmental status of embryos is graded based on their morphology. In contrast to blastocysts, degenerative embryos appear morphologically retarded in their development. These embryos do not exhibit a distinct inner cell mass and have no blastocoele. Using this system, single nucleotide polymorphisms in several genes have been shown to be associated with fertilization and blastocyst rates [8-10]. The aim of this study was to characterize transcriptomic differences between IVF blastocysts and degenerative embryos. This is the first direct and comprehensive comparison between in vitro-produced embryos with distinct morphological phenotypes. Although remarkably similar gene expression profiles were found between blastocysts and degenerative embryos, a total of 67 differentially expressed transcripts were identified. Results of this study may help elucidate transcriptomic changes associated with abnormal development in mammalian species and facilitate improvement of assisted reproductive technologies.

## Results

### Global transcriptomic changes in degenerative embryos compared to blastocysts

**Figure 1:**
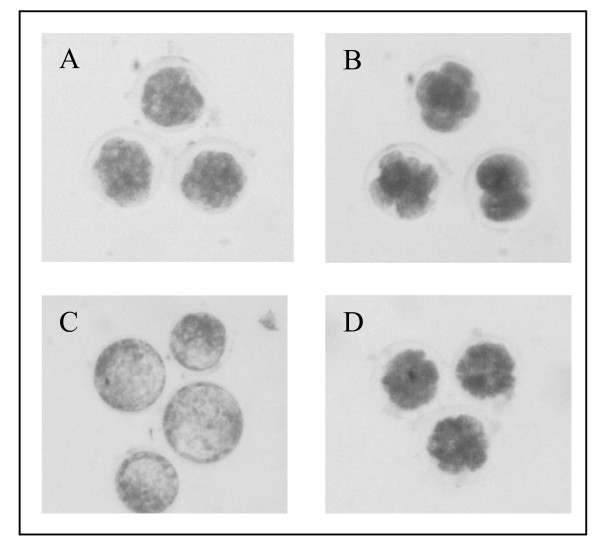
**Examples of morphological stage grading used in this study**. Putative zygotes were cultured until day 5, when embryos were examined for evidence of compaction. Embryos that showed compaction by this time were classified as compacted morula (A) while embryos that did not exhibited compaction or attained 16-32 cells were classified as "early degenerative" (B). Compacted morulas were further cultured until day 8 when they were evaluated for presence of blastocoele. Embryos that showed distinct inner cell mass and blastocoele were classified as "blastocysts" (C) and embryos that did not properly complete transition from morula to blastocyst were classified as "late degenerative" (D). Transcriptomic profiles of blastocysts (C) and late degenerative embryos (D) were compared in this study.

**Figure 2:**
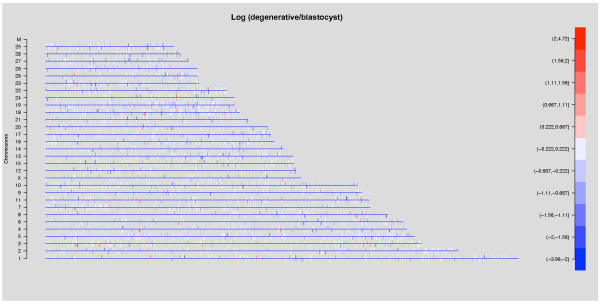
**Global change in transcriptomes of degenerative embryos**. Differences between the mean log2 transformed expressions of degenerative embryos and blastocysts plotted along each chromosome. 18 transcripts on the "Y" chromosome could not be plotted because there was no physical location information available for the "Y" chromosome from the current genome assembly. Each vertical bar on the chromosomes represents one transcript and was colored according to the expression difference. Bars above the axis were transcripts on the forward strand while bars below the axis were transcripts on the reverse strand. Blue color represents lower expression in degenerative embryos compared with blastocysts while red color represents higher expression in degenerative embryos, as indicated by the scale bar on the right.

In order to characterize global transcriptomic changes of degenerative embryos concurrent with their abnormal embryonic development, a comparative microarray experiment was designed. Because the amount of RNA present in a single embryo is rather limited, three independent pools each consisting of 20 embryos were constructed for blastocysts and degenerative embryos (Figure 1). Total RNA was extracted from each pool of embryos and subjected to linear amplification [11] before standard microarray labeling and hybridization were performed on a GeneChip Bovine Genome Array [12]. A total of 14,509 and 14,411 transcripts were detected as expressed in blastocysts and degenerative embryos, respectively. Interestingly, Pearson's correlation between averaged gene expression of blastocysts and degenerative embryos was 0.986, suggesting a high similarity between their gene expression profiles. To visualize transcriptomic changes with respect to physical locations, change in gene expression of degenerative embryos as compared to blastocysts was plotted along each chromosome (Figure 2). Notably, the majority of transcripts showed little or no difference between blastocysts and degenerative embryos. In addition, differentially expressed genes scattered across all chromosomes without any apparent pattern. Taken together, these results suggest that although there were distinct phenotypic outcomes, little change had occurred in the transcriptome of degenerative embryos as compared to blastocysts.

### Identification and clustering of differentially expressed genes

**Figure 3:**
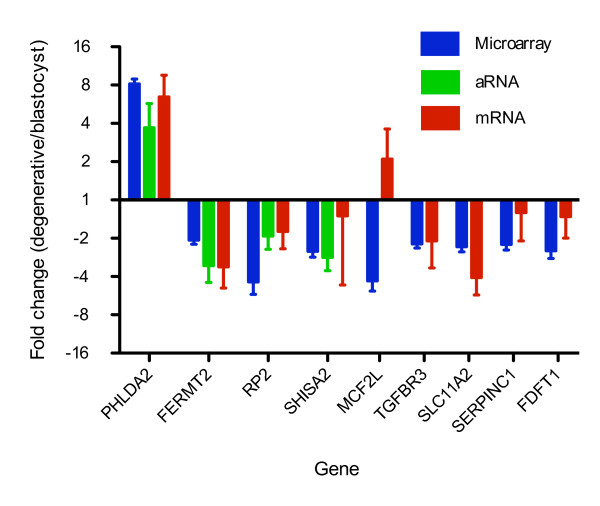
**Real-time RT-PCR validation of microarray results**. All expressions were normalized to *GAPDH *in the same RNA sample, analyzed by the 2^-ΔΔCt ^method [44]. Data is shown as (Mean +/- SEM) fold changes. Upregulation in degenerative embryos is represented by bars above the x axis while downregulation in degenerative embryos is represented by bars below the x axis. For genes *PHLDA2*, *FERMT2*, *RP2*, and *SHISA2*, real-time RT-PCR was carried out in three amplified aRNA samples and two unamplified mRNA samples for each of blastocysts and degenerative embryos (Additional file 1). For genes *MCF2L*, *TGFBR3*, *SLC11A2*, *SERPINC1*, and *FDFT1*, real-time RT-PCR was performed in a different set of three unamplified mRNA samples for each of blastocysts and degenerative embryos (Additional file 1).

Although there was little change to the global transcriptome of degenerative embryos, significance analysis of microarrays (SAM) identified 67 (false discovery rate (FDR) = 0.15) transcripts differentially expressed between blastocysts and degenerative embryos, of which 33 showed at least a two-fold difference (Table 1). Among these 33 differentially-expressed transcripts, three were upregulated in degenerative embryos whereas the remaining transcripts were downregulated. In order to validate the microarray results, *PHLDA2*, *FERMT2*, *RP2*, *SHISA2*, *MCF2L*, *TGFBR3*, *SLC11A2*, *SERPINC1 *and *FDFT1 *were chosen for quantitative gene expression using real-time RT-PCR (see Methods for gene selection criteria). Different sources of RNA were used to accomplish biological replications. For *PHLDA2*, *FERMT2*, *RP2*, and *SHISA2*, we carried out real-time RT-PCR in the same amplified RNA (aRNA) samples used for microarrays as well as RNA samples from four independently constructed embryo pools (Additional file 1). For the remaining five genes, another independent set of six RNA pools (three for each of blastocysts and degenerative embryos) were used. Importantly, although magnitude and variability of fold change differed slightly between the different sources of RNA and quantitation methods (microarray and real-time RT-PCR), the same trends observed in the microarrays were also observed in the real-time RT-PCR. For eight of the nine examined genes, expression differences between degenerative embryos and blastocysts were confirmed in the real-time RT-PCR (Figure 3), underscoring the validity of our experimental procedures and data analysis. For example, *PHLDA2 *was upregulated eight-fold in degenerative embryos as observed with microarray whereas real-time RT-PCR using unamplified mRNA detected a six-fold upregulation. Nonetheless, different sensitivity and bias were expected for different quantitation methods. The exception to microarray confirmation by real-time RT-PCR was *MCF2L*, a gene whose variability in expression was notably high. In degenerative embryos, *MCF2L *appeared to be downregulated in the microarray results yet appeared upregulated in the real-time RT-PCR analysis.

**Table 1:** Transcripts differentially expressed by at least two-fold in degenerative embryos as compared to blastocysts (FDR <= 0.15)

Gene symbol^1^	Gene name	Fold change	P value^2^
Upregulated			
*PHLDA2*	pleckstrin homology-like domain, family A, member 2	8.18	0.00002
*LOC540268*	hypothetical LOC540268	4.19	0.00008
*C14H8ORF70*	chromosome 8 open reading frame 70 ortholog	3.01	0.00017
Downregulated			
*CMBL*	carboxymethylenebutenolidase homolog (Pseudomonas)	2.16	0.00004
*CTNS*	cystinosis, nephropathic	2.09	0.00005
*TNNC2*	troponin C type 2 (fast)	3.47	0.00015
*TGFBR3*	transforming growth factor, beta receptor III	2.22	0.00022
*DAPP1*	dual adaptor of phosphotyrosine and 3-phosphoinositides	3.01	0.00024
*FERMT2*	fermitin family homolog 2 (Drosophila)	2.07	0.00026
*PECR*	peroxisomal trans-2-enoyl-CoA reductase	2.14	0.00031
*SLC11A2*^3^	solute carrier family 11 (proton-coupled divalent metal ion transporters), member 2	2.33	0.00035
*Bt.19510.2.A1_at*	transcribed locus	2.33	0.00036
*Bt.21611.1.S1_at*	transcribed locus	2.32	0.00037
*SHISA2*	shisa homolog 2 (Xenopus laevis)	2.54	0.00039
*Bt.22693.1.A1_at*	transcribed locus	7.31	0.00042
*MGC157372*	hypothetical LOC614796	2.34	0.00046
*MCF2L*	MCF.2 cell line derived transforming sequence-like	4.32	0.00050
*SLC10A1*	solute carrier family 10 (sodium/bile acid cotransporter family), member 1	12.67	0.00055
*SERPINC1*	serpin peptidase inhibitor, clade C (antithrombin), member 1	2.23	0.00058
*CYP11A1*	cytochrome P450, family 11, subfamily A, polypeptide 1	2.14	0.00063
*Bt.6523.1.A1_at*	transcribed locus	2.49	0.00077
*LOC521943*	similar to hCG1788238	3.41	0.00078
*SLC11A2*^3^	solute carrier family 11 (proton-coupled divalent metal ion transporters), member 2	2.19	0.00079
*Bt.17542.1.S1_at*	transcribed locus	2.43	0.00081
*Bt.27347.1.A1_at*	transcribed locus	3.05	0.00082
*PTPRK*	protein tyrosine phosphatase, receptor type, K	4.04	0.00087
*Bt.19644.1.A1_at*	transcribed locus	3.20	0.00088
*FDFT1*	farnesyl-diphosphate farnesyltransferase 1	2.52	0.00089
*CYP51*	cytochrome P450, family 51, subfamily A, polypeptide 1	3.48	0.00094
*RP2*	retinitis pigmentosa 2 (X-linked recessive)	4.41	0.00095
*LOC790609*	similar to aminoacylase 1	2.45	0.00098
*LOC513587*	similar to UPF0474 protein C5orf41	4.33	0.00116
*SLC25A21*	solute carrier family 25 (mitochondrial oxodicarboxylate carrier), member 21	2.75	0.00119

^1 ^Transcripts without annotations were identified by probe set ID.

^2 ^Raw p values from significance analysis of microarray (SAM), transcripts were ordered according to their p values.

^3 ^The gene SLC11A2 is represented by multiple probe sets on the Bovine Genome Array

**Figure 4:**
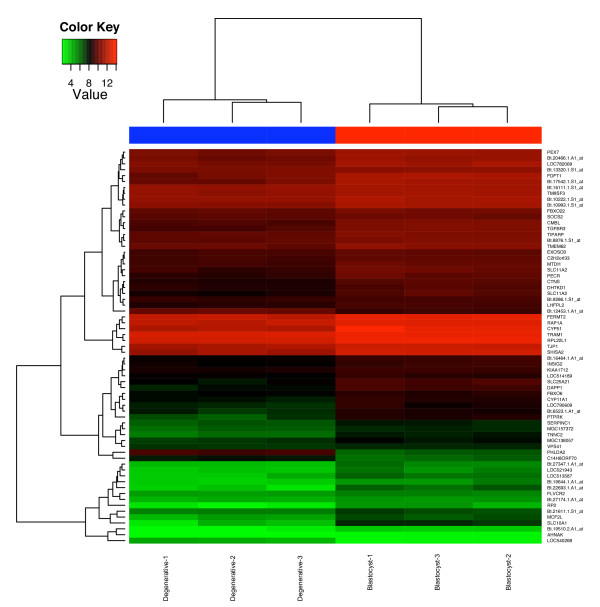
**Clustering of expression of candidate genes involved in early embryonic development**. Expression levels for 67 differentially expressed genes for the six samples were hierarchically clustered and shown in a heatmap. Level of expression was represented by color scale from green (low) to red (high), as indicated by a scale bar in the upper left corner. Dendrograms of distances were also shown for genes (left) and samples (top). Names of samples and genes were indicated on the bottom and right, respectively. For genes without annotation, probe set IDs were shown.

To qualitatively explore patterns of co-regulation of the 67 differentially-expressed genes [13], expression profiles of these genes were clustered and visualized in a heatmap (Figure 4). Notably, *FERMT2 *(*MIG2*), *RAP1A*, and *TJP1 *(*ZO1*) showed closely clustered expression levels and patterns, and all of them have been shown to be involved in a pathway related to cell adhesion functions [14-16], suggesting that at least for this signaling pathway, systematic expression alteration had occurred in degenerative embryos compared to blastocysts.

### Signaling pathways associated with abnormality of embryonic development

To further understand transcriptomic changes that have occurred in degenerative embryos at a systems level, Gene Set Enrichment Analysis (GSEA) [17] and gene ontology (GO) enrichment analysis were carried out. GSEA identifies *a priori *defined signaling pathways whose member genes show expression correlated with developmental status of embryos. A collection of 649 signaling pathways in the MsigDB database curated from various sources [17] were interrogated. GSEA at 25% FDR revealed five pathways enriched in degenerative embryos and four pathways enriched in blastocysts (Table 2). In addition, GO terms were also tested for overrepresentation in differentially expressed genes using a hypergeometric test, and five GO terms were significant at 25% FDR (Table 2). Remarkably, both GSEA and GO enrichment pointed to a significant association of steroid biosynthesis and cell communication ('Small GTPase mediated signal transduction' is a child of the GO term 'cell communication') processes with developmental status (Table 2). This evidence strongly suggested involvement of these pathways in abnormal embryonic development.

**Table 2:** Gene Set Enrichment Analysis (GSEA) and GO enrichment analysis results with FDR = 0.25

GSEA pathways	**Sample size (n/m)**^1^	P value	GO categories	Count/Expected count	P value
Enriched in degenerative embryos^2^			Biological process		
Nuclear receptors	23/40	<0.001	Cholesterol metabolic process	10/1.4	<0.001
Monoamine GPCRs	20/33	<0.001	Steroid biosynthetic process	8/1.5	<0.001
Cell communication	76/138	<0.001	Small GTPase mediated signal transduction	18/8.3	<0.001
GPCRs class A rhodopsin like	78/185	<0.001	Cellular component		
Cytokine pathway	18/22	0.003	Endoplasmic reticulum membrane	18/8.4	0.002
Enriched in normal embryos^3^			Molecular function		
Biosynthesis of steroids	19/24	<0.001	Transferase activity, transferring alkyl or aryl groups	6/1.4	0.002
Met pathway	28/36	0.003			
N-glycan biosynthesis	34/42	0.005			
Linoleic acid metabolism	17/31	0.011			

^1 ^n = number of genes in the analyzed dataset; m = number of genes in the original gene set

^2 ^Enrichment in degenerative embryo means significantly more genes in this gene set showed higher expression in degenerative embryos

^3 ^Enrichment in blastocysts means significantly more genes in this gene set showed higher expression in blastocysts

## Discussion

In the present study we report the transcriptomic profiling of blastocysts and degenerative embryos and identification of candidate genes and pathways involved in early embryonic development. While global gene expression in blastocysts and degenerative embryos was largely similar, 67 (33 with greater than 2 fold difference) transcripts were significantly different between these two groups of embryos. In addition, several signaling pathways were found to be altered in degenerative embryos as compared to blastocysts. Although there has been a number of reports on dynamics of transcriptomes in IVF embryos [5,6], this study, to the best of our knowledge, reports the first direct and comprehensive comparison between blastocysts and degenerative embryos produced by IVF. We believe transcriptomic alteration characterized through this comparison could provide insights into mechanisms of early embryonic development and may help identify biomarkers for growth defect in IVF and for infertility in cattle.

### Validity of the experimental design and microarray analysis

Pooling of embryos and amplification of RNA in this study were necessitated by scarcity of RNA present in embryos [5,6]. These approaches have been well documented in the literature [5,18,19]. In this study, we pooled a relatively large number (n = 20) of independent embryos in each pool to achieve a sufficient accuracy of biological pooling. In order to validate the results of microarray experiment and analysis, a total of 16 different pools (eight for each of blastocysts and degenerative embryos) from two different sources of RNA were used as biological replications in the real-time RT-PCR experiments (Figure 3 and Additional File 1). Importantly, eight out of the nine differentially-expressed genes identified through the microarray experiment and SAM were validated in real-time RT-PCR, testifying to the validity of the experimental design and the analysis used in this study.

It is possible that some of the degenerative embryos could be in different developmental stages because of the three-day time window of embryo collection. However, we prefer not to narrow down this window into one or two days because shorter times would result in substantial disturbance to embryo culture and mischaracterization of the two groups of embryos. Thus, we assume that there is some variation in gene expression within the degenerative embryos and that this variation would be reduced by pooling the embryos for expression analysis. Poor synchronization of embryos and integrity of RNA extracted from embryos could potentially introduce errors to the experiment. RNA integrity was checked before every major step in the microarray experiment to ensure sample quality. Moreover, although pooling of embryos removes variation between individual embryos from expression measurements, correlations between gene expression of pools can be used to assess synchronization in the same group of embryos and identify outliers due to compromise of RNA integrity. In fact, correlations of gene expression between samples were 0.979-0.995 within the group of blastocysts and 0.951-0.990 among degenerative embryos. This is an evidence of synchronization and integrity of the biological samples used in this study.

A comparison between gene expression profiles of embryos in this study with embryos at the same developmental stage from other studies would also indicate whether RNA and data quality was compromised in our samples. In fact, high correlations between gene expression profiles of our samples and those of Kues *et al*. [6] using the same microarray platform were observed. The correlations between our IVF blastocysts and those of Kues *at al*. were 0.942-0.970, and the correlations between our degenerative embryos and IVF blastocysts of Kues *et al*. were 0.925-0.957. Collectively, these results suggest that blastocysts and degenerative embryos used in the present study were largely synchronized to the same stage.

### Biologically sensible results

We identified 67 differentially-expressed transcripts and several candidate pathways associated with abnormal early embryonic development. The identification of previously known candidate genes or pathways is also an important aspect of microarray experiments. Interestingly, a number of genes and pathways identified in this study fall into this category of biologically sensible results. *PHLDA2 *(also known as *TSSC3*) was found to be upregulated in degenerative compared to blastocysts by both microarray and real-time RT-PCR (Table 1 and Figure 3). *PHLDA2 *is an apoptosis-related gene that maps to a paternally-imprinted region involved in cancer development [20]. The imprinting status of bovine *PHLDA2 *is not yet known. However, two known imprinted genes *H19 *and *IGF2 *are located nearby on bovine chromosome 29 [21,22], and the whole conserved cluster is imprinted in human and mouse [20,23]. Interestingly, overexpression of *PHLDA2 *in mice caused placental growth retardation [24] while *PHLDA2 *knock-out mice showed placental overgrowth [25], indicating that proper *PHLDA2 *expression is required for normal placental growth. Thus, our result offers further support for the importance of tightly regulated expression of *PHLDA2 *and may indicate its involvement in earlier stages of development.

Another differentially-regulated gene in degenerative embryos versus blastocysts is *TGFBR3*, one of the three types of receptors for TGF beta and one that regulates ligand binding of TGF beta to type I and type II receptors [26]. In addition, although not meeting our FDR cutoff, the TGF beta signaling pathway was significantly (p = 0.046) associated with the developmental status of embryos. Collectively, these two lines of evidence suggest an important role of TGF beta signaling pathway in normal embryonic development, which has been reported in other studies [27]. Cell communication and steroid biosynthesis pathways identified by both GSEA and GO enrichment analyses are of particular interest (Table 2) and their roles in early embryonic development have been studied extensively. For example, gap junctions and cell communication have been well documented to have profound influence on early embryonic development [28,29], while several steroid hormones are required for transition from morula into blastocyst stage [30,31].

### Small change, large effect

Although the phenotypic outcomes of degenerative embryos and blastocysts were distinct, we did not observe dramatic transcriptomic changes differentiating these two groups of embryos. The correlation between gene expression of blastocysts and degenerative embryos was relatively high (r = 0.986). Among the 67 differentially- expressed transcripts, 33 were changed by more than two fold while only eight of them differed by more than four-fold, and two of them by eight-fold. One may argue that the sample size in this study is not large enough to detect small changes, so that many genuine differentially-expressed genes were missed. This is true for most microarray experiments, which normally do not involve many samples. However, our pooling strategy that reduces variation between samples presumably should alleviate this problem. Indeed, about half (34/67) of the differentially-expressed genes were less than two-fold different. These results suggest that small transcriptomic changes can lead to the distinct phenotype observed in the degenerative embryos and that the high degree of similarity observed between degenerative embryos and blastocysts was a results of true effects rather than of insufficient experimental power.

The ability to detect differentially expressed genes can also be limited by completeness of transcripts manufactured on the microarray platform. The Affymetrix Bovine Genome Array has 24,128 probe sets representing over 23,000 bovine transcripts. Contents of the array were based on GenBank and UniGene databases. Although it is possible that some transcripts exclusively expressed during early development are not represented on the array, it is unlikely that too many transcripts are missed.

The size of the differences in expression may be a specific characteristic of these genes, yet small changes in gene expression can lead to pronounced phenotypic change. For example, silencing by microRNAs has been shown to be less than two-fold [32,33], yet they have been suggested to regulate a wide range of developmental processes to a large degree. Thus, our results suggest that small transcriptomic changes could lead to the abnormal development of degenerative embryos.

### Influence of in vitro culture

There have been several reports comparing genome wide mRNA profiles between IVF and in vivo blastocysts [7,34]. These studies demonstrated that expressions of a number of genes were changed in IVF blastocysts as compared to embryos produced in vivo. Identification of genes affecting quality of IVF embryos due to culture system is undoubtedly important. Nevertheless, comparison between blastocysts and degenerative embryos in this study is also important because only 30%-35% zygotes can successfully develop to blastocyst stage *in vitro*, a large source of economic loss. There are likely to be various reasons for unsuccessful development *in vitro *but genetics seems to play an important role [8,10]. Embryos cultured *in vitro *are in a unified environment; therefore significant differential expressions detected are likely to be associated with developmental defect rather than culture system. In fact, we compared our list of differentially expressed genes to the 200 genes that showed expression differences between IVF embryos and embryos produced *in vivo *by artificial insemination [34]. Importantly, among the 67 differentially expressed genes identified in our study, only one gene (DAPP1) showed in vitro/in vivo difference [34]. This result suggests that the differentially expressed genes identified in this study were likely due to developmental defect rather than culture system.

## Conclusions

In summary, we found that the transcriptome of degenerative embryos was largely unchanged as compared to their blastocysts counterparts, yet there was a relatively small number of candidate genes that displayed differential expression between the two groups of embryos. We also found several signaling and metabolic pathways associated with bovine early embryonic development. Importantly, the results presented provide useful information in conceiving future experiments aiming at the mechanistic understanding of early embryonic development as well as improving current assisted reproductive technology. There is a growing body of studies reporting the use of the bovine as a suitable model for human infertility and embryonic development [3,4]. As such, genes and pathways associated with early embryonic development identified in this study can be utilized to investigate similar traits in other mammalian species.

## Methods

### In vitro fertilization and sample preparation

Ovaries from mature cows were collected at a local abattoir and immediately followed by aspiration of oocytes from antral follicles (> 2-6 mm). Oocytes were processed, incubated in maturation media, and allowed to mature for 24 hours. Mature oocytes were combined with bull semen adjusted to a final concentration of 1 × 10^6^/mL sperm. Frozen thawed bull semen was Percoll separated as described previously [35] using a discontinued 45%-95% gradient. Putative zygotes were cultured for 120 hours (5 days) before they were evaluated for evidence of compaction or cell coalescence. On day 5 of development in vitro (fertilization = day 0) embryos were viewed via light microscopy to assess morphological development (Figure 1). Embryos that exhibited compaction (cellular coalescence) were classified as compacted morula. Embryos that have not attained 16-32 cells and that did not exhibit compaction were classified as early degenerate. Early degenerative embryos might include a range of cellular development from 2 cell (initial cleavage) up to 8-16 non-compacting cells and were removed from the culture and excluded from further analysis. Embryos showing evidence of compaction were cultured for additional 72 hours (day 8 of development) until they were morphologically graded as blastocysts or degenerative. On day 8, embryos exhibiting a distinct inner cell mass and obvious blastocoele were classified as blastocysts. Embryos that did not properly transition from morula to blastocyst were classified as late degenerative embryos (Figure 1). These two groups of embryos were subjected to microarray and subsequent analysis. Blastocysts and late degenerative embryos were collected and stored in RNAlater (Ambion, TX) to preserve RNA integrity. Embryo culture conditions and media were as described [10]. Briefly, putative zyotes were cultured in syntheic oviductal fluid (Biowhittaker, Walkersburg, MD) supplemented with 0.22 mM sodium pyruvate, 25 ug/mL gentamicin sulfate and 8 mg/mL essentially fatty acid-free BSA. Three pools consisting of 20 randomly sampled blastocysts or degenerative embryos were created. Embryos were produced from 5 bulls and 57 cows. Each pool contained embryos from 5 - 12 cows and 3 - 5 bulls. Total RNA was extracted from pools of embryos using RNaqueous Micro (Ambion, TX) and quality controlled using a RNA6000 PicoChip (Agilent Technologies, CA). The PicoChip was analyzed on an Agilent 2100 Bioanalyzer (Additional file 2) according to manufacturer's instructions. Approximately 100 ng of total RNA was purified from a pool 20 embryos.

### Linear amplification and labeling of complementary RNA (cRNA)

Due to the limited amount of RNA present in embryos, a two-round linear amplification was employed to amplify and label whole polyadenylated pools of RNA [11] using the MessageAmp II aRNA amplification kit (Ambion, TX). Briefly, the first round of amplification was achieved by priming cDNA synthesis with a T7 promoter tagged poly-dT primer and in vitro transcription by T7 polymerase with unlabeled NTPs. Purified first-round aRNA was quality checked and then subjected to the second round amplification and labeling with biotinlyted UTP following manufacturer's protocol. In fact, for all Affymetrix arrays this second round of amplification and labeling must be performed, and total RNA is used as input [12].

### Array hybridization and data acquisition

A total of 15 ug of the biotin-labeled cRNA was fragmented and hybridized to GeneChip Bovine Genome Array (Affymetrix, CA). After staining and washing, microarrays were scanned using a GC3000 7G scanner at the University of Wisconsin Biotechnology Center Gene Expression Center. Raw data was acquired by GeneChip^® ^Operating Software v1.4 (GCOS) and stored as .CEL files. Data from Kues *et al*. [6] was downloaded from GEO database at NCBI with the accession number GSE12327 as .CEL files.

### Analysis of microarray data

All data analysis was carried out using Bioconductor 2.3 [36] packages implemented with R 2.8.1 [37]. Microarray expression intensities were preprocessed using the 'GCRMA' (v2.14.1) [38] package in Bioconductor, which corrected backgrounds based on calculated affinities of probe sequences, quantile-quantile normalized intensities, and summarized expressions of probe sets through median-polish and log2 transformed expression values. MAS5 detection calls [39] were used to qualitatively classify transcript presence. Transcripts that were called "P" (present) in at least two out of the three samples were classified as "Expressed" whereas transcripts called "A" (Absent) in all three samples were classified as "Not expressed". Unclassified transcripts represented genes whose expressions were at the detection limit or at extremely low levels.

There are a total of 24,128 probe sets on the Bovine Genome Array. Probe sets that represent spike-in controls or did not vary across all samples were removed, leaving a total of 18,946 probe sets. Significance Analysis of Microarrays (SAM) [40] was used to identify differentially-expressed probe sets by the 'siggenes' (v1.16.0) package in Bioconductor, with the False Discovery Rate (FDR) controlled at 15%. Differentially-expressed genes were hierarchically clustered and visualized using functions from the 'gplots' (v2.6.0) package in R.

Gene Set Enrichment Analysis (GSEA) [17] was carried out using a desktop version of GSEA which queried 'canonical pathways' (v2.5) in the MsigDB database http://www.broad.mit.edu/gsea/msigdb/index.jsp. It is a collection of 649 gene sets curated from various sources including KEGG, GenMAPP, and gene ontology among others. Gene sets were permuted 5000 times to estimate FDRs for enrichments. In addition, 352 unique differentially-expressed Entrez genes having gene ontology (GO) annotations were identified by setting FDR to 25% in SAM. Enrichments for GO terms were tested by a hypergeometric test ('GOstats' package v2.8.0) with respect to 6598 unique GO annotated Entrez genes on the bovine array. Hypergeometric p values were corrected using the Benjamini-Hochberg method [41] to control FDR at 0.25 for GO enrichments. Two additional filters were applied to minimize false positives: 1) GO categories with fewer than 20 genes were dropped; 2) when a significant GO category is a parent of (contains) another significant GO category, only the child was considered. Enrichments with FDR = 0.25 [42] were presented.

### Real-time RT-PCR

To validate results obtained by microarrays, nine genes (*PHLDA2*, *FERMT2*, *RP2*, *SHISA2*, *MCF2L*, *TGFBR3*, *SLC11A2*, *SERPINC1*, *FDFT1*) found differentially expressed by microarrays were tested using real-time RT-PCR. Genes were chosen to represent a wide range of fold changes. *FERMT2*, *SHISA2*, *TGFBR3*, *SLC11A2*, *SERPINC1*, and *FDFT1 *were changed between two- and four-fold. *RP2 *and *MCF2L *were changed between four- and eight-folds. And *PHLDA2 *was changed over eight-fold. Five additional pools of blastocysts and degenerative embryos were constructed from which RNA was extracted as described above. cDNA was synthesized from first round aRNA in the microarray experiment and the independent mRNA samples using the iScript cDNA synthesis kit (Bio-Rad Laboratories, CA). Dilutions of cDNA were used as template for real-time PCR using iQ SYBR Green Supermix (Bio-Rad Laboratories, CA). The reference gene *GAPDH *was amplified as an endogenous control. Importantly, the expression of *GAPDH *on microarrays was largely invariable across samples. To establish the stability of *GAPDH*, two additional reference gene *RPLP0 *(ribosomal protein large P0) and ACTB (actin, beta) were chosen and stability of *GAPDH *was M = 0.4 as calculated using the Vandesompele method [43]. To achieve sufficient biological replication of samples, the genes were divided into two groups and tested separately using different sources of RNA. For *PHLDA2*, *FERMT2*, *RP2*, and *SHISA2*, three aRNA samples used for microarray and two independent mRNA pools were tested for each of blastocysts and degenerative embryos (Additional file 1). For the remaining five genes, three different mRNA pools were tested for each of blastocysts and degenerative embryos (Additional file 1). Relative gene expressions were calculated using the 2^-ΔΔCt ^method [44]. All primers used are listed in Table 3.

**Table 3:** Primer sequences in real- time RT-PCR reactions and products' sizes

Gene	Primer	Sequence (5' - 3')	Amplicon (bp)
*GAPDH*	Forward	TGCCCAGAATATCATCCC	134
	Reverse	AGGTCAGATCCACAACAG	
*PHLDA2*	Forward	CCTAAGTCCCACGGCGAATC	109
	Reverse	CTATATCCTTGCCCTGGTCAGC	
*FERMT2*	Forward	GATTAGGATGGACGCCAGCAC	128
	Reverse	AGGACAACCGTACTTCATCTGC	
*SHISA2*	Forward	GCGGCTGCGACAACGATC	130
	Reverse	ATGAAGGCGACAAACACTGACC	
*RP2*	Forward	AAGCACCTGACTTCCTTCCTC	119
	Reverse	CTTGGTCCCTTTGAATGTCTCG	
*TGFBR3*	Forward	TCGCTGGATGCCTCAATG	140
	Reverse	ATCTGTGGAGTAATTGGAATCG	
*MCF2L*	Forward	TGAGCCTGGAGGGATACG	110
	Reverse	GCCATCGTTGTCCTCAGG	
*SLC11A2*	Forward	TGCAGTGGTCAGCGTAGC	111
	Reverse	TTAGAGATGCTTACCGTGTGC	
*SERPINC1*	Forward	AAGTCCAGGCTCCCAGGTATTG	142
	Reverse	GCGAACGACCAGCGATGC	
*FDFT1*	Forward	GGTCACCCTGATGATGGATGC	139
	Reverse	CCTGATGGTGGAGATGATCTGC	
*ACTB*	Forward	AGGCCAACCGTGAGAAGATGAC	100
	Reverse	CCAGAGGCATACAGGGACAGC	
*RPLP0*	Forward	GACAATGGCAGCATCTAC	198
	Reverse	GAAGGTGTAATCAGTCTC	

## Authors' contributions

WH and HK designed the study and wrote the manuscript. WH performed the microarray and real-time PCR experiments. WH and BY analyzed the data. All authors read and approved the final paper.

## Supplementary Material

Additional file 1 **RNA extracted/amplified from pools of embryos and RNA used for real time RT-PCR**. Sources of RNA used for the real time RT-PCR validation of microarray results. Click here for file

Additional file 2 **Representative gel like images of RNA from blastocysts and degenerative embryos**. Two representative images of RNA extracted from blastocyst and degenerative embryos. The RNA was analyzed by a RNA6000 PicoChip on BioAnalyzer 2001. Click here for file
