# Supplementary material for: Transcriptomic profiling of bovine IVF embryos revealed candidate genes and pathways involved in early embryonic development
Source: BMC Genomics. 2010 Jan 11;11:23. doi: 10.1186/1471-2164-11-23 (PMC2824717; doi:10.1186/1471-2164-11-23)
Supplement: Additional file 1 — RNA extracted/amplified from pools of embryos and RNA used for real time RT-PCR. Sources of RNA used for the real time RT-PCR validation of microarray results. [file 1471-2164-11-23-S1.DOC]

Additional file 1

RNA extracted/amplified from pools of embryos and RNA used for real time RT-PCR

|  | Blastocysts | Degenerative embryos |
| --- | --- | --- |
| **RNA extracted/amplified from embryo pools** |  |  |
| aRNA used for microarray | B1^1^, B2, B3 | D1^2^, D2, D3 |
| mRNA | B4, B5, B6, B7, B8 | D4, D5, D6, D7, D8 |
| **RNA used for real time RT-PCR** |  |  |
| PHLDA2, FERMT2, RP2 and SHISA2 | B1, B2, B3, B4, B5 | D1, D2, D3, D4, D5 |
| MCF2L, TGFBR3, SLC11A2, SERPINC1 and FDFT1 | B6, B7, B8 | D6, D7, D8 |

1 Bs indicate pools of “blastocysts” and each number represents a unique pool. For example, N1, N2 and N3 are three normal embryo pools linearly amplified and used for microarrays. In addition to N1, N2 and N3, N4-8 are five pools whose total RNAs were extracted and used for real time RT-PCR of genes as indicated by the last two rows of the table.

2 Ds indicate pools of “degenerative embryos” and each number represents a unique pool.
