# Supplementary material for: Transcriptomic profiling of bovine IVF embryos revealed candidate genes and pathways involved in early embryonic development
Source: BMC Genomics. 2010 Jan 11;11:23. doi: 10.1186/1471-2164-11-23 (PMC2824717; doi:10.1186/1471-2164-11-23)

Additional file 2. Representative gel like images of RNA from blastocysts and degenerative embryos

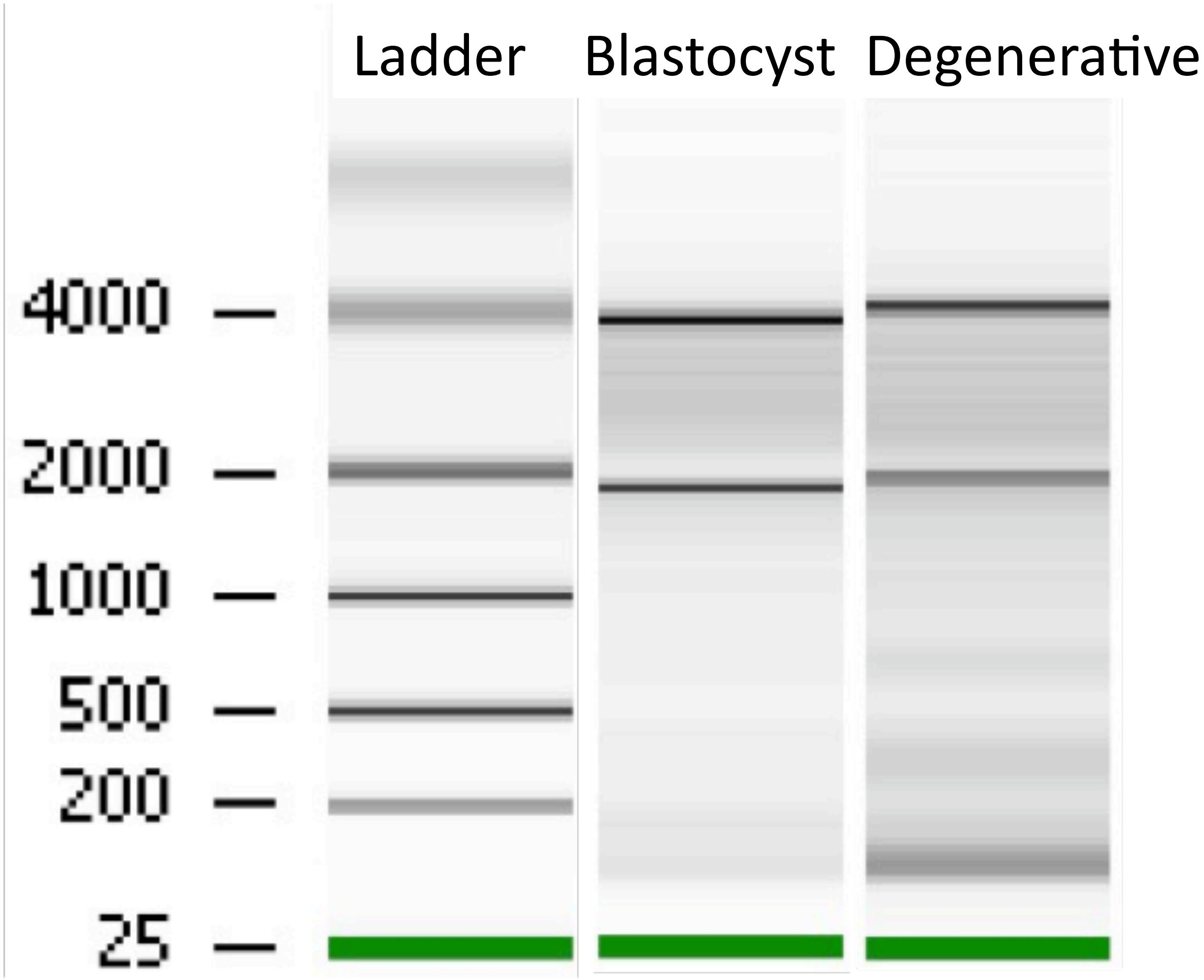

Supplement: Additional file 2 — Representative gel like images of RNA from blastocysts and degenerative embryos. Two representative images of RNA extracted from blastocyst and degenerative embryos. The RNA was analyzed by a RNA6000 PicoChip on BioAnalyzer 2001. [file 1471-2164-11-23-S2.PDF]
